# Root Architecture and Functional Traits of Spring Wheat Under Contrasting Water Regimes

**DOI:** 10.3389/fpls.2020.581140

**Published:** 2020-11-11

**Authors:** Nidia Brunel-Saldias, Juan Pedro Ferrio, Abdelhalim Elazab, Massiel Orellana, Alejandro del Pozo

**Affiliations:** ^1^Centro de Mejoramiento Genético y Fenómica Vegetal, Facultad de Ciencias Agrarias, Universidad de Talca, Talca, Chile; ^2^Fundacion Agencia Aragonesa para la Investigacion y el Desarrollo (ARAID), Zaragoza, Spain; ^3^Department of Forest Resources, Agrifood Research and Technology Center of Aragón (CITA), Zaragoza, Spain; ^4^Department of Botany, Faculty of Natural Sciences and Oceanography, University of Concepción, Concepción, Chile

**Keywords:** root biomass, root weight density, water status, water isotopic signature, water use efficiency

## Abstract

Wheat roots are known to play an important role in the yield performance under water-limited (WL) conditions. Three consecutive year trials (2015, 2016, and 2017) were conducted in a glasshouse in 160 cm length tubes on a set of spring wheat (*Triticum aestivum* L.) genotypes under contrasting water regimes (1) to assess genotypic variability in root weight density (RWD) distribution in the soil profile, biomass partitioning, and total water used; and (2) to determine the oxygen and hydrogen isotopic signatures of plant and soil water in order to evaluate the contribution of shallow and deep soil water to plant water uptake and the evaporative enrichment of these isotopes in the leaf as a surrogate for plant transpiration. In the 2015 trial under well-watered (WW) conditions, the aerial biomass (AB) was not significantly different among 15 wheat genotypes, while the total root biomass and the RWD distribution in the soil profile were significantly different. In the 2016 and 2017 trials, a subset of five genotypes from the 2015 trial was grown under WW and WL regimes. The water deficit significantly reduced AB only in 2016. The water regimes did not significantly affect the root biomass and root biomass distribution in the soil depths for both the 2016 and 2017 trials. The study results highlighted that under a WL regime, the production of thinner roots with low biomass is more beneficial for increasing the water uptake than the production of large thick roots. The models applied to estimate the relative contribution of the plant’s primary water sources (shallow or deep soil water) showed large interindividual variability in soil, and plant water isotopic composition resulted in large uncertainties in the model estimates. On the other side, the combined information of root architecture and the leaf stable isotope signatures could explain plant water status.

## Introduction

The rainfed agriculture of the Mediterranean regions is suffering from water shortages, which strongly limit current yields of wheat and other cereals (Richards, 1996; Chairi et al., 2020). Water deficiency in these regions is predicted to increment in the future because climatic change is reported to reduce precipitation and raise evapotranspiration (Carvalho et al., 2014; del Pozo et al., 2019a). Furthermore, competition for the currently available water resources will also increase due to population growth and human activities (e.g., industry, mining, and tourism) (Araus, 2004; Araus et al., 2013). The current trends in population growth and the consequent increases in food needs make the Mediterranean regions food-insecure, particularly in the Mediterranean basin, where at least 215 million are at risk of qualitative and quantitative food insecurity (Prosperi et al., 2014).

The genetic gain in grain yield (GY) of spring wheat cultivars developed during the green revolution has been high in favorable environments (del Pozo et al., 2014, 2019b), but this has not been the case in drought-stressed environments where the productivity of modern cultivars is even lower than traditional cultivars (Elazab, 2015; Crespo-Herrera et al., 2017). This is due to the fact that breeding strategies used during the green revolution were mainly focused on maximizing the yield potential under optimal growing conditions (Araus et al., 2002). Moreover, previous breeding efforts for increasing GY in drought-prone Mediterranean regions relied on selecting yield *per se*, distinguished by low heritability and high genotype × environment interactions (Jackson et al., 1996; Elazab, 2015).

Root traits play an important role in drought tolerance and cereals’ yield performance under water-limited (WL) conditions (Blum, 2009; Comas et al., 2013). The ability of plant roots to uptake water and nutrients from a given depth of the soil profile relies on root distributional traits, such as the root length and weight (Tinker and Nye, 2000; Li et al., 2006; Elazab et al., 2012, 2016; Carvalho et al., 2014; Zhao et al., 2018). Root weight density (RWD) is often used in root studies (Fageria, 2004; Zhang et al., 2009; Elazab et al., 2012, 2016; Shen et al., 2013; Wang et al., 2014; Ali et al., 2018; Zhao et al., 2018) to describe the root weight allocation within a volume of the soil profile. Previous studies showed contradictory effects of water regimes on RWD; for instance, Xue et al. (2003) and Ali et al. (2018) reported increases in RWD with water supply, whereas Elazab et al. (2012, 2016) reported no changes in RWD by water deficits, or even increased under these conditions. Overall, previous studies (Elazab et al., 2012, 2016) reported the RWD to associate with more water uptake under optimal and water-deficit conditions.

Studies of root growth responses to water deficit in breeding programs have received much less attention compared to the drought-adaptive traits of shoots (Lynch, 2007; Richards et al., 2010; Palta et al., 2011; Shen et al., 2013; Wang et al., 2014; Lü et al., 2015), and it is still a challenging subject for research due to: (1) the absence of easy and efficient root selection techniques under field conditions that could be applied to study root phenotyping traits in a large number of plants and in soil depths of more than 100 cm; (2) root growth is affected by several factors such as soil type, density of planting, water and fertilizer applications, and tillage practices; (3) previous reports have generally studied the effects of only one factor on the root system (mainly soil water or nitrogen content) while ignoring the interaction of the studied factor with other factors in the soil; (4) the plant growth and morphology of shoot and root systems is known to be modified by the volume available for root growth in pot or tube experiments, independent of stresses, and thus, the results of these experiments need field validation; and (5) the genetics of many root traits (e.g., complex polygenic traits like total root system size), including patterns of inheritance and heritability, have not been well understood until now.

During the past two decades, the stable oxygen isotope composition (δ^18^O) of plant tissues has been widely studied because it integrates the evaporative conditions throughout the crop cycle (Barbour et al., 2000). The δ^18^O of leaf water is isotopically enriched during transpiration (Barbour and Farquhar, 2000). Therefore, it has been used as a proxy method for measuring plant transpiration as well as for detecting genotypic differences in stomatal conductance (*gs*) in wheat (Barbour et al., 2000; Sheshshayee et al., 2005; Cernusak et al., 2007, 2009; Ferrio et al., 2007). The δ^18^O measured in plant tissues is known to reflect variation in (1) the isotopic composition of source water, (2) evaporative enrichment in leaves due to transpiration, and (3) biochemical fractionation during the synthesis of organic matter (Barbour et al., 2005; Ferrio, 2005; Elazab et al., 2015).

The effect of environment on transpiration and evaporation also drives leaf water evaporative ^2^H enrichment in the plant similar to δ^18^O (Cernusak et al., 2016; Sánchez-Bragado et al., 2019). Therefore, the plant δ^2^H in plant tissue is influenced by both *gs* and the effects of climate on transpiration (Sternberg et al., 1984; Cernusak et al., 2016). A high correlation of δ^18^O with δ^2^H in plant tissue may elucidate the source (i.e., water) and environmental effects (Epstein et al., 1977), while the lack of correlation could be due to additional hydrogen or oxygen isotope fractionation effect (Barbour et al., 2005; Sánchez-Bragado et al., 2019).

That plant root system plays an essential role in water uptake and water movement across the soil–plant–atmosphere continuum (SPAC) (Guo et al., 2016). Most studies have determined plant water uptake by traditional methods, such as phenological or root system traits (Qiu et al., 2008; Yang et al., 2010; Nosalewicz and Lipiec, 2014; Guan et al., 2015; Zhao et al., 2018). However, these methods do not efficiently identify the water source (Ehleringer and Dawson, 1992; Zhao et al., 2018). Thus, root water absorption patterns cannot be indicated only by root system distribution (Asbjornsen et al., 2007; Guo et al., 2016).

The stable isotope technique is increasingly used to understand SPAC’s water movement (Sulzman et al., 2007; Guo et al., 2016). The isotopic fractionation occurs in physical transport processes, and thus, the isotopic signatures of various water sources tend to be different (Schwendenmann et al., 2015; Guo et al., 2016). The analyses of the oxygen and hydrogen isotopic signatures of water as natural tracers provide an efficient, accurate, and non-destructive tool for discovering the plant’s primary water sources (Dawson and Simonin, 2011; Schwendenmann et al., 2015; Ma and Song, 2016). The technique relies on the fact that the isotope signature of xylem water is a mixture of different water sources accessible for the plant (Dawson and Ehleringer, 1991). No oxygen or hydrogen isotope fractionation occurs during root water uptake or the transportation process in the stem xylem in the majority of plants, except for a few coastal wetland species and few woody xerophytes that fractionate the stable hydrogen isotope but not the stable oxygen isotope during root water uptake (Wershaw et al., 1966; Zimmerman et al., 1967; White et al., 1984; Lin et al., 1993; Mensforth et al., 1994; Dawson et al., 2002; Ellsworth and Williams, 2007). It is also assumed that the fractionation of oxygen and hydrogen isotopes follows the same pattern during evaporation (Thorburn and Walker, 1993). Thus, the natural vertical gradients of hydrogen and oxygen stable isotopic signatures in soil water give the same information about plant water uptake depth from the soil profile (Plamboeck et al., 1999). Therefore, the plant’s primary water sources can be determined by comparing the isotopic ratios of all potential water sources (such as precipitation or irrigation, soil water from varying depths, groundwater), which vary widely in isotopic composition, with the isotopic ratio of water extracted from the xylem representing a weighted average of soil water uptake by functional roots (Ehleringer and Dawson, 1992; Eggemeyer et al., 2009; Zhou et al., 2017).

This work investigated genotypic variability in root distribution in a set of spring wheat cultivars and advanced lines with contrasting yield and tolerance to water deficit, growing in polyvinyl chloride (PVC) tubes under well-watered (WW) and WL regimes in glasshouse conditions. The objectives were to (1) assess root growth and distribution, aerial biomass (AB), and total water used (WU) of the wheat genotypes under two contrasting water regimes; and (2) determine the oxygen and hydrogen isotopic signatures (δ^18^O, δ^2^H) of plant and soil water to evaluate the contribution of shallow and deep soil water to plant water uptake and the evaporative enrichment of these isotopes in the leaf as a surrogate for plant transpiration.

## Materials and Methods

### Plant Material, Growing Conditions, and Experimental Design

**FIGURE 1:**
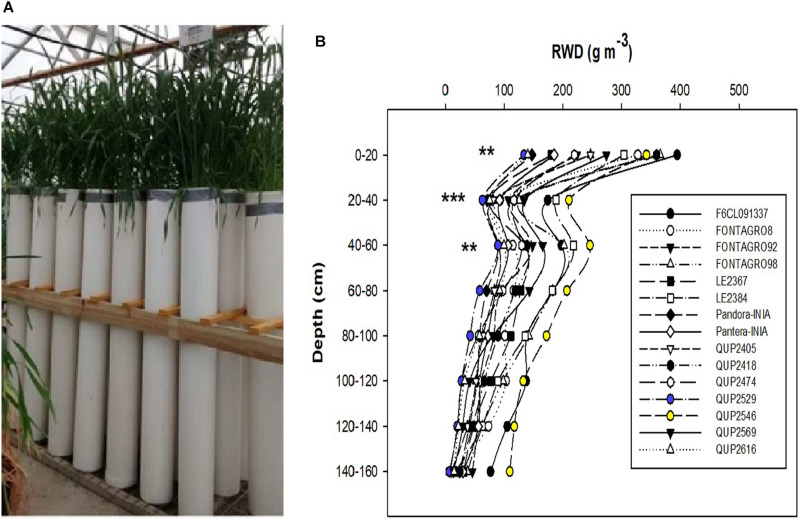
**(A)** Wheat genotypes growing in PVC tubes of 16 cm diameter and 160 cm length in a heated glasshouse, and **(B)** distribution of root biomass in soil depth of the 15 genotypes grown under well-watered regime in 2015. **P ≤ 0.01 and ***P ≤ 0.001 are obtained from ANOVA test of the different genotypes at the different soil column depths.

Three glasshouse trials were carried out at the Plant Breeding and Phenomic Center (35°24′19″S; 71°37′59″W), Talca University, Talca, Chile, from 2015 to 2017. In 2015, a set of 13 advanced lines and two cultivars of spring wheat (*Triticum aestivum* L.) with contrasting performance under water deficit (Table 1) was chosen from a set of 384 genotypes (del Pozo et al., 2016, 2020). Plants were sown in PVC tubes of 16 cm diameter and 160 cm length in a heated glasshouse (Figure 1A) on 10 August. The glasshouse had natural lighting and a heating system (climatic details presented in Supplementary Table S3). One seed was sown in each tube, which was filled with a 1:1:1 mixture of perlite, sand, and organic soil mixture (Anasac, Chile). Before filling the tubes with the substrate mixture, several substrate mixture samples were weighted and dried at 60°C for 48 h to calculate the substrate’s water content. The day before sowing, the tubes containing the substrate were saturated by water and left for drainage. At sowing, the tubes were weighed, the substrate’s dry weight was subtracted from the saturated substrate’s weight after drainage, and the water holding capacity per tube was calculated (equivalent to the field capacity). A 3-cm layer of perlite aggregates was added on the tube top surface to prevent evaporation. Before weekly irrigation, tubes were weighed, and the amount of WU by the plants due to transpiration was calculated. For the WW regime, the tubes were maintained at 100% of the tube water holding capacity during the whole growth cycle by adding the amount of transpired water. Plants were fertilized with medium strength (70%) Hoagland nutrient solution (Hoagland and Arnon, 1938) using 100, 250, 250, and 300 ml at 15, 30, 45, and 60 days after sowing, respectively.

**TABLE 1:** Set of wheat genotypes tolerant or susceptible to water stress according to the yield tolerance index (YTI), total aerial biomass (AB), total root biomass (RB), and root to shoot ratio (R:S) in 2015.

Genotype	YTI^1^	Tolerance to stress	AB (g)	RB (g)	R:S
QUP2418	0.67	Tolerant	55.15 a	3.87 bcd	0.07 bcd
QUP2546	0.56	Tolerant	61.30 a	6.21 d	0.09 bcd
**FONTAGRO8**	**0.52**	**Tolerant**	38.05 a	3.80 bcd	0.10 bcd
LE2367	0.47	Tolerant	56.98 a	3.18 abc	0.06 abc
**QUP2529**	**0.44**	**Tolerant**	49.46 a	1.77 a	0.03 a
QUP2474	0.44	Tolerant	56.32 a	3.16 abc	0.06 abc
FONTAGRO92	0.37	Intermediate	39.77 a	3.31 abc	0.08 bcd
QUP2405	0.36	Intermediate	59.47 a	2.66 ab	0.04 ab
QUP2616	0.33	Intermediate	50.30 a	2.16 ab	0.04 ab
LE2384	0.31	Intermediate	44.70 a	4.81 bcd	0.11 d
**Pantera-INIA**	**0.38**	**Intermediate**	59.36 a	2.76 ab	0.05 ab
Pandora-INIA	0.26	Susceptible	60.67 a	2.23 ab	0.04 a
**QUP2569**	**0.21**	**Susceptible**	53.94 a	3.55 abc	0.06 abc
F6CL091337	0.16	Susceptible	52.32 a	5.81 cd	0.11 d
**FONTAGRO98**	**0.15**	**Susceptible**	42.83 a	4.92 bcd	0.11 d

*Means followed by different letters were significantly different (P ≤ 0.05) by Tukey’s HSD test. The genotypes used in the 2016 and 2017 trials are indicated in bold. ^1^According to del Pozo et al. (2016), higher values of yield tolerance index (YTI) indicate more tolerance to water stress. YTI = Y_WS_Y_FI_/$\bar{Y}$_FI_^2^, where Y_WS_ and Y_FI_ are the genotype yield under water stress (Cauquenes) and full-irrigation conditions (Santa Rosa), respectively, in the 2012 growing season, and $\bar{Y}$_FI_ is the mean yield of all genotypes under full-irrigation conditions.*

In the 2016 and 2017 trials, a subset of five genotypes (Table 1) was evaluated in the same tubes and soil mixture used in 2015 under WW and WL regimes. The WW regime was applied similar to the 2015 trials, while the WL regime was applied by adding 50% of the amount of transpired water for the WW regime tubes until the tubes reached 50% of the initial tube water holding capacity (around heading) and then tubes were maintained at 50% until harvest. The water regimes commenced when the flag leaves were fully expanded (Zadoks stage Z41) in 2016 and at tillering (Z26) in 2017 and finished at anthesis (Z69) (Zadoks et al., 1974). Sowing dates were 12 September in 2016 and 11 May in 2017. In 2016, the amount of Hoagland nutrient solution applied was similar to the 2015 trial (total 900 ml), while it was increased in 2017 where it was applied at four dosages of 200, 500, 500, and 500 ml at 15, 30, 45, 60 and 75 days after sowing, respectively.

In 2015, the experiment was set up as a randomized complete block design in three blocks and a total number of 45 tubes in the 2015 trial (15 genotypes × 3 replicates). In 2016 and 2017, the experiments were arranged as a factorial experiment in a randomized complete block design in three replicates with a total number of 30 tubes (5 genotypes × 2 water regimes × 3 replicates).

### Evaluation of Aerial and Root Biomass, Water Use, and Root Weight Density

Plants were harvested at anthesis (Z69) for the 3-year trials. The growing periods were 108 days in 2015 (harvested on 26 November), 79 days in 2016 (harvested on 30 November), and 126 days in 2017 (harvested on 14 September). For AB and root biomass (RB) determination, the plants were separated into shoots and roots at harvest.

The tube’s total volume was sectioned every 20 cm (representing profile layers at depths of 0–20, 20–40, 40–60, 60–80, 80–100, 100–120, 120–140, 140–160), and each layer was weighed to determine the volumetric content of water. The roots were carefully washed, and then both the roots and the AB were rapidly dried in an oven at 60°C for 72 h. The root to shoot ratio (R:S) was calculated as the ratio of RB to AB. The plants’ total amount of water transpired during the growing cycle, or the total WU by the plant due to transpiration in each tube, was determined. The water use efficiency (WUE, g L^–1^) was calculated as (Tambussi et al., 2007):

$$\text{WUE}=\frac{\text{AB}}{\text{WU}}$$

Root dry weight was measured for each of the soil sections. The RWD of each soil section (RWD_*L*_, g m^–3^) was calculated as (Carvalho, 2009; Elazab et al., 2012, 2016):

$${\text{RWD}}_{\text{L}}=\frac{{\text{RB}}_{\text{L}}}{\pi \times R^{2}\times L}$$

where RB_*L*_ = root dry weight in the soil section (g), *R* = tube radius (0.08 m), and *L* = length of the soil section (0.20 m long).

### Determination of Isotope Composition in Plant and Soil Water

In 2017, the oxygen and hydrogen isotopic signatures (δ^18^O, δ^2^H) of the plant and soil water were used to determine (1) the contribution of shallow and deep soil water to plant water uptake and (2) the evaporative enrichment in the leaf, as a surrogate for plant transpiration. Different samples were collected during harvest, including soil samples at two depths (0–20 and 140–160 cm), the plant root collar, and the flag leaf. Samples were placed in 15 ml airtight plastic tubes and rapidly frozen at −24°C until distillation. Water extraction was performed at the Laboratory of Plant Physiology (University of Concepción) using cryogenic vacuum distillation, adapting the method described in Palacio et al. (2014). Briefly, tubes were put in a heated water bath (80°C) and connected with Ultra-Torr^TM^ unions (Swagelok Company, Solon, OH, United States) to a vacuum system (−760 in Hg), in series with U-shaped collector tubes cooled with liquid nitrogen. After the extraction time (2 h), the trapped water was transferred into 2 ml vials and stored at 4°C until analysis. For each sample, water content (as a percentage of fresh weight) of the distilled material (soil, plant tissue) was determined from the weight change before and after distillation. Water isotopic composition (δ^18^O, δ^2^H) was determined with cavity ring-down spectroscopy (CRDS) in a Picarro L2120-i isotopic water analyzer (Picarro Inc., Sunnyvale, CA, United States), coupled to a high-precision vaporizer (A0211), at the Scientific-Technical Services of the University of Lleida (Lleida, Spain). The potential spectral interference caused by organic contaminants in the water extract was corrected, according to Martín-Gómez et al. (2015). Isotopic enrichment of mean lamina leaf water above the source water (Δ^18^O, Δ^2^H, in %) was calculated according to Ferrio et al. (2009):

$$\Delta =\left(\delta_{\text{L}}-\delta_{\text{S}}\right)/\left(1+\delta_{\text{S}}\right)$$

where δ_*L*_ and δ_*S*_ stand for the isotopic signatures of leaf water and source water, respectively. Following Barnard et al. (2007), a section of the root collar and basal stem was considered here to be representative of the source water.

### Data Analysis

Data were analyzed through a one-way analysis of variance (ANOVA) in 2015. For both the 2016 and 2017 trials, a combined ANOVA could not be conducted because the homogeneity of variance test (Levene’s test) was significant. Thus, two-way ANOVAs were run for the 2016 and 2017 trials separately using the general linear model (GLM) procedure to calculate the effects of genotypes (G), water regime (W), and G × W interaction.

Mean separation of genotypes was performed by Tukey’s honestly significant difference (HSD) test (*P* ≤ 0.05). Relationships between the studied traits were analyzed using Pearson linear correlation, where the correlations were constructed from the genotypic means within each water regime. Data were analyzed using the SPSS 24 statistical package (IBM Crop) and R 3.5.2 (R Core Team, 2018).

The relative contribution of different water sources to plant water uptake was estimated using the SIAR package for R, which solves Bayesian mixing models for stable isotopic data (Parnell et al., 2010). The model uses multiple isotope values of “consumers” (individual values of δ^18^O, δ^2^H of stem water) and sources (deep and shallow soil water, mean plus standard deviation) as inputs. The function “siarmcmcdirichletv4” was used, in which the output is calculated on a population basis, classifying individual plants into different groups (genotypes), setting the number of iterations, and burning and thinning successively to 5,000,000, 500,000, and 15,000.

## Results

### Plant Biomass and Water Use

According to the results of the previous field trial conducted at the rainfed (Cauquenes) and full-irrigation (Santa Rosa) sites in 2012, the yield tolerance index (YTI) classified the studied genotypes into tolerant, intermediate, and susceptible to water deficits (Table 1). In 2015, the genotypes growing under the WW regime showed no significant differences in AB, but the RB exhibited significant differences, where the genotypes QUP2529 and QUP2546 presented the lowest and highest RB, respectively (Table 1). Also, R:S exhibits significant differences among genotypes; the lowest value was for genotype QUP2529 and highest for FONTAGRO98, F6CL091337, and LE2384 (Table 1).

For the 2016 trial (Table 2), the genotypic effects were significant for all the studied traits except for the WUE. The water regimes application significantly affected all studied traits except RB, where the WL regime reduced AB by 17%, increased R:S by 15%, decreased WU by 29%, and increased WUE by 17% compared with the WW regime genotype mean. The genotype × water regime (G × W) interaction was significant for R:S, WU, and WUE.

**TABLE 2:** Aerial biomass (AB, g), root biomass (RB, g), root:shoot ratio (R:S), water use (WU, L), and water use efficiency (WUE, g L^–1^) of five wheat genotypes grown under well-watered (WW) and water-limited (WL) conditions–2016 trial.

Genotype		AB	RB	R:S	WU	WUE
Pantera-INIA		23.95 a	2.41 a	0.10 a	9.35 ab	2.94 a
QUP2569		31.05 b	4.13 c	0.13 b	12.16 c	2.92 a
FONTAGRO98		24.90 a	3.06 ab	0.12 b	9.86 b	2.88 a
QUP2529		31.62 b	3.25 b	0.10 a	12.47 c	2.80 a
FONTAGRO8		20.75 a	2.42 a	0.12 ab	8.84 a	2.70 a
WW		28.95	3.15	0.11	12.33	2.59
WL		23.95	2.96	0.13	8.74	3.10

**ANOVA**						

**SV**	**DF**	**F value**				

R	2	0.73	1.02	1.85	1.90	0.96
G	4	19.57***	15.95***	7.16***	67.45***	1.29
W	1	27.49***	1.36	15.35***	390.47***	44.90***
G × W	4	1.00	2.46	5.79**	9.58***	3.94*
Error	48					

Analysis of variance for the effect of genotype (G), water regime (W), and their interaction (G × W) is also shown. Genotype means followed by different letters were significantly different (P ≤ 0.05) by Tukey’s HSD test. *P ≤ 0.05; **P ≤ 0.01; and ***P ≤ 0.001. The genotype mean value represents the average of 2 water regimes × 3 replicates (n = 6 per genotype). SV, source of variation; DF, degrees of freedom, R, replicates.

For the 2017 trial (Table 3), significant genotypic differences were detected for all studied traits except for WUE. The water regimes application significantly affected the WU and WUE, where the WL regime decreased WU by 51% and increased WUE by 52% compared with the WW regime genotype mean. Also, the G × W was significant only for AB and WUE.

**TABLE 3:** Aerial biomass (AB, g), root biomass (RB, g), root:shoot ratio (R:S), water use (WU, L), and water use efficiency (WUE, g L^–1^) under well-watered (WW) and water-limited (WL) conditions–2017 trial.

Genotype		AB	RB	R:S	WU	WUE
Pantera-INIA		44.57 a	2.17 a	0.05 a	10.59 a	5.26 a
QUP2569		52.30 b	4.04 c	0.08 b	11.93 b	5.10 a
FONTAGRO98		49.42 ab	3.02 ab	0.06 ab	11.76 b	5.29 a
QUP2529		52.63 b	3.23 bc	0.06 ab	11.41 b	5.76 a
FONTAGRO8		51.05b	3.38 bc	0.07 ab	11.74 b	5.07 a
WW		49.97	3.39	0.07	15.54	3.44
WL		50.02	2.95	0.06	7.43	7.16

***ANOVA***						

***SV***	***DF***	***F value***				

R	2	1.97	0.46	0.84	1.08	2.43
G	4	2.97*	7.58***	2.85*	5.73**	2.61
W	1	0.00	3.94	2.62	1,647.79***	589.29***
G × W	4	8.73***	0.91	1.67	1.44	12.46***
Error	48					

Analysis of variance for the effect of genotype (G), water regime (W), and their interaction (G × W) is also shown. Genotype means followed by different letters were significantly different (P ≤ 0.05) by Tukey’s HSD test. *P ≤ 0.05; **P ≤ 0.01; and ***P ≤ 0.001. The genotype mean value represents the average of 2 water regimes × 3 replicates (n = 6 per genotype). SV, source of variation; DF, degrees of freedom; R, replicates.

For both the 2016 and 2017 trials, the genotype QUP2569 showed the highest values for most studied traits, while the cv. Pantera-INIA showed the opposite response (Tables 2, 3).

### Distribution of Roots and Soil Water Content in the Soil Profile

In 2015, the maximum root depth was similar among the 15 genotypes, but the distribution of roots in depth expressed as RWD differed significantly at the upper (0–20, 20–40, and 40–60 cm) layers; the genotype QUP2546 showed higher RWD in almost all soil depths and the opposite response for the genotype QUP2529 (Figure 1B).

**FIGURE 2:**
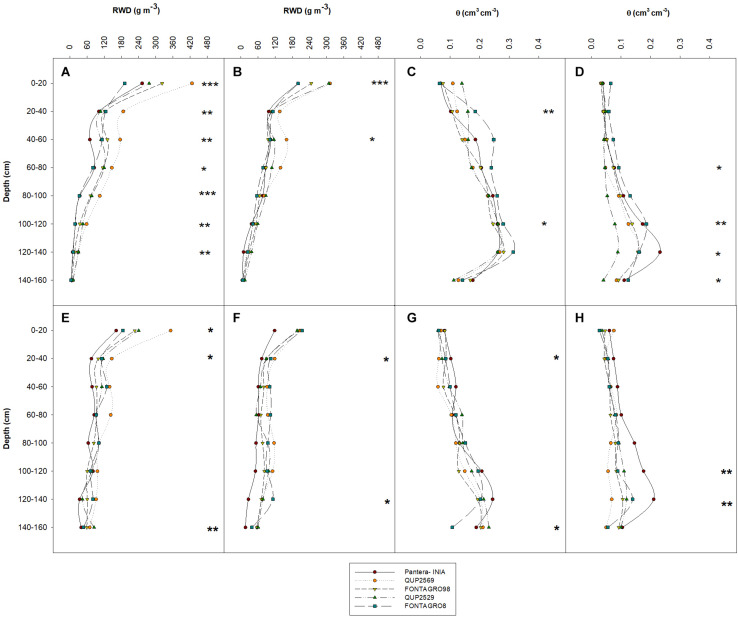
Distribution of root biomass **(A,B,E,F)** and soil water content **(C,D,G,H)** in depth at anthesis of five spring wheat genotypes with contrasting tolerance to water deficit, grown under well-watered **(A,E,C,G)** and water-limited conditions **(B,F,D,H)**, in tubes of 160 cm length in a glasshouse in 2016 **(A–D)** and 2017 **(E–H)**. *P ≤ 0.05, **P ≤ 0.01, and ***P ≤ 0.001 are obtained from ANOVA test of the different genotypes at the different soil column depths.

The water regimes did not change RWD distribution depth in both the 2016 and 2017 trials except for the upper (40–60 cm) layer in the 2017 trial, where the RWD under WL decreased by 26% compared with the WW regime genotype mean (Supplementary Tables S1, S2). Significant genotypic differences in RWD were detected for most soil layers (except for the 140–160 cm) under the WW regime and for the upper layers (0–20 and 40–60 cm) under the WL regime in 2016 and the upper (0–20 and 20–40 cm) and lower (120–140 and 140–160 cm) soil layers of both water regimes in 2017 (Figures 2A–F and Supplementary Tables S1, S2). The genotype QUP2569 tended to show the highest RWD under WL and WW regimes in both trials, and cv. Pantera-INIA tended to show the opposite response.

At harvest, the residual soil water content (SWC) under the WL regime at all soil layers was significantly lower than under the WW regime (SWC decreased by 40–72 and 22–58% in the WL regime in 2016 and 2017, respectively, relative to the WW regime genotype mean) (Supplementary Tables S1, S2). In 2016, significant genotypic differences in SWC were detected in the upper (20–40 cm) and lower–middle (100–120 cm) soil depths under the WW regime (Supplementary Table S1 and Figure 2C). Also, genotypic differences were observed under the WL regime, in the middle (60–80 and 100–120 cm) and lowest (120–140 and 140–160 cm) soil layers (Supplementary Table S1 and Figure 2D). Similarly, significant genotypic differences were detected in 2017, particularly in the upper (20–40 cm) and lowest (140–160 cm) soil layers under the WW regime (Supplementary Table S2 and Figure 2G) and for the lower–middle (100–120 cm) and the lowest (120–140 cm) soil layers under the WL regime (Supplementary Table S2 and Figure 2H). Under both the WL and WW regimes, cv. Pantera-INIA exhibited the highest values of SWC at most of the studied soil layers, while the genotype QUP2569 showed the opposite response at most of the studied soil layers (Supplementary Table S2).

In 2016, the AB and WU showed significant and positive correlations with RWD under both water regimes, but this was not the case in 2017, except for WU under the WL regime (Table 4). Furthermore, AB and WU showed strong and significant positive correlations in 2016 in both water regimes.

**TABLE 4:** Pearson correlation coefficients of aerial biomass (AB, g) and water use (WU, L) with root weight density (RWD, g m^–3^).

	WU	RWD1	RWD2	RWD3	RWD4	RWD5	RWD6	RWD7	RWD8
**2016**									
**WW**									
AB	0.92**	0.59*	0.25	0.44	0.73**	0.79**	0.80**	0.80**	0.67**
WU	1.00	0.68**	0.45	0.5	0.80**	0.76**	0.78**	0.83**	0.70**
**WL**									
AB	0.93**	0.90**	0.45	0.69**	0.64*	0.57*	0.41	0.53*	0.13
WU	1.00	0.88**	0.34	0.60*	0.64*	0.57*	0.38	0.52*	0.22
**2017**									
**WW**									
AB	0.13	0.18	0.27	0.32	−0.02	0.32	0.47	0.43	0
WU	1.00	0.22	0.32	0.22	0.19	−0.23	−0.23	0.26	−0.32
**WL**									
AB	0.37	0.37	−0.06	−0.01	−0.1	0.25	0.2	0.28	0.47
WU	1.00	0.68**	0.73**	0.59*	0.61*	0.81**	0.69**	0.69**	0.69**

The correlations were calculated within each water regime using the genotype values of the three replicates (n = 15). WW, well-watered, WL, water-limited. Probabilities (*P ≤ 0.05; **P ≤ 0.01) are shown. The number following the acronym of the trait refers to the soil column depth where the trait was measured: 1 refers to 0–20 cm; 2 refers to 20–40 cm; 3 refers to 40–60 cm; 4 refers to 60–80 cm; 5 refers to 80–100 cm; 6 refers to 100–120 cm; 7 refers to 120–140 cm; 8 refers to 140–160 cm. Bold numbers represent significant correlations.

### Soil Water Content at Harvest and Water Isotope Composition

Shallow (0–20 cm) and deep (140–160 cm) SWC (WC%) at harvest was significantly reduced under the WL regime by 42 and 37%, respectively, compared with the WW regime genotype mean (Table 5). The genotypic effect for soil WC was not significant. The stem and leaf water content (WC%, FW) were similar in both water regimes, but the genotypic effect was significant for the leaf water content (Table 5).

**TABLE 5:** Analysis of variance for the effect of treatment, genotype and their interaction on water content (WC,% of fresh weight), water isotope composition (δ^18^O, δ^2^H) in soil depths (shallow, deep) and plant tissues (basal stem, leaf), and leaf water isotopic enrichment above source water (Δ^18^O, Δ^2^H).

Genotype		Shallow soil WC (%)	Deep soil WC (%)	Shallow soil δ^18^O (%)	Deep soil δ^18^O (%)	Shallow soil δ^2^H (%)	Deep soil δ^2^H (%)	Stem WC (% FW)	Stem δ^18^O (%)	Stem δ^2^H (%)	Leaf WC (% FW)	Leaf δ^18^O (%)	Leaf δ^2^H (%)	Leaf Δ^18^O (%)	Leaf Δ^2^H (%)
Pantera-INIA		12.20 a	25.60 a	−10.66 a	−7.81 a	−83.49 a	−65.45 a	68.00 a	−10.22 a	−72.98 a	72.60 ab	15.28 b	27.60 ab	25.77 b	108.60 ab
QUP2569		9.00 a	24.20 a	−10.10 a	−8.80 a	−84.02 a	−56.36 a	66.10 a	−11.26 a	−79.90 a	73.40 ab	27.21 c	56.50 c	38.92 c	148.45 c
FONTAGRO98		9.30 a	23.00 a	−13.01 a	−9.07 a	−88.81 a	−56.77 a	64.50 a	−8.72 a	−66.76 a	71.40 c	15.57 b	31.81 b	22.94 ab	97.56 a
QUP2529		12.70 a	21.20 a	−8.99 a	−8.76 a	−78.14 a	−71.37 a	68.50 a	−9.08 a	−71.25 a	75.20 a	7.69 a	9.43 a	17.22 a	87.23 a
FONTAGRO8		10.10 a	28.20 a	−9.60 a	−8.42 a	−78.78 a	−63.93 a	67.20 a	−9.18 a	−74.58 a	73.10 ab	18.90 b	39.12 bc	27.31 b	115.12 b
WW		13.40	30.20	−11.31	−9.38	−82.96	−66.77	66.00	−11.41	−80.67	73.70	16.00	32.96	28.25	124.43
WL		7.80	19.10	−9.72	−7.73	−82.54	−61.30	67.70	−8.31	−67.01	72.70	17.79	32.26	25.72	102.33

**ANOVA**															

**SV**	**DF**	**F value**													

R	2	0.61	0.80	0.05	2.03	0.24	1.82	0.44	1.00	1.11	1.54	0.06	0.27	0.73	2.44
G	4	1.92	0.92	1.22	1.20	0.32	1.57	1.34	0.42	0.32	3.65*	18.81***	13.24***	16.40***	7.90***
W	1	36.91***	21.38***	1.61	5.58*	0.00	1.71	1.95	5.32*	4.66*	2.25	1.37	0.03	1.85	8.36**
G × W	4	1.82	1.29	0.85	0.48	0.69	0.72	1.51	0.67	0.10	1.95	2.45	2.34	1.15	1.51
Error	10														

Data from the 2017 trial. Genotype means followed by different letters were significantly different (P ≤ 0.05) by Tukey’s HSD test. *P ≤ 0.05; **P ≤ 0.01; and ***P ≤ 0.001. WW, well-watered plants; WL, water-limited plants; G, genotype; W, water regime; G × W, genotype by water regime interaction; SV, source of variation; DF, degrees of freedom; R, replicates.

The analysis of the stable isotopes indicated that (1) the WL regime significantly increased the stable isotope composition of the deep soil δ^18^O, stem δ^18^O, and stem δ^2^H, by 1.65, 3.1, and 13.66%, respectively, while it decreased the leaf Δ^2^H by 22.1%, compared with the WW regime genotype mean; (2) the genotypic effects were significant for both leaf oxygen and hydrogen stable isotope compositions (leaf δ^18^O and δ^2^H) and enrichments (leaf Δ^18^O and leaf Δ^2^H) (Table 5); and (3) the G × W effects were not significant for any of the studied traits (Table 5). Overall, the genotype QUP2529 showed the highest leaf WC values and the lowest leaf δ^18^O, leaf δ^2^H, leaf Δ^18^O, and leaf Δ^2^H (Table 5).

**FIGURE 3:**
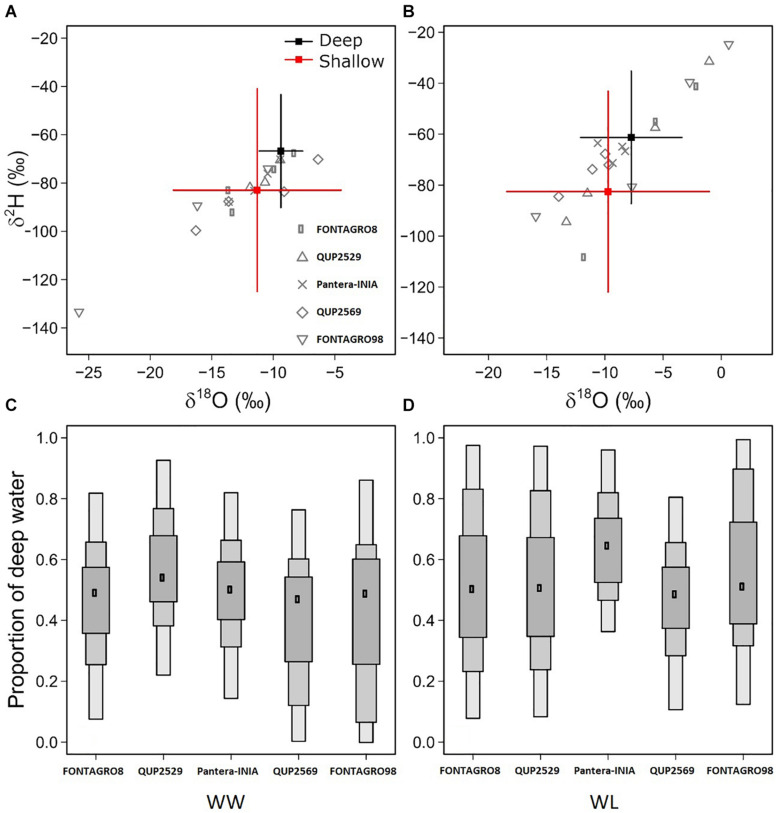
Quantification of the proportion of water uptake from deep soil depths in the 2017 trial, based on Bayesian isotope mixing models. The biplots show the oxygen and hydrogen isotope composition (δ^18^O, δ^2^H) in basal stem water (as a surrogate of xylem water), and the reference values (sources) of deep and shallow soil water, for WW **(A)** and WL **(B)** plants. The box plot indicates the mode and the 95, 75, and 50% credible intervals for the estimated proportion of root collar water derived from deep soil depths, in WW **(C)** and WL **(D)** plants.

The Bayesian mixing models were applied to estimate the relative contribution of different soil profile depths to plant water uptake. Overall, the models showed large interindividual variability in soil and plant water isotopic composition (Figures 3A,B), resulting in large uncertainties in the model estimates (Figures 3C,D).

## Discussion

### Plant Biomass Allocation, Water Use, and Water Use Efficiency Responses to Water Regimes

Despite the significant effect of the applied water regimes on water use (WU) and the SWC at harvest in the whole soil profile, the WL regime application did not decrease RB or RWD distribution significantly in the 2016 and 2017 trials, except in the upper soil depth (40–60 cm) in 2017 (Tables 2, 3 and Supplementary Tables S1, S2). These results agree with studies supporting the idea that the biomass allocated to roots may remain unaffected by water deficits (Sharp and Davies, 1979; Barraclough, 1989; Sacks et al., 1997; Elazab et al., 2016).

The decrease in AB and the increase in the R:S and WUE are well-known plants’ responses to water deficit (Tambussi et al., 2007; Elazab et al., 2012, 2016). Results of the 2016 trial agreed with previous studies because the increase in the R:S under the WL regime was due to the reduction in AB (reduced by 18%) rather than the reduction in RB (Table 2), and thus, WUE increased by 17% compared with the WW regime genotype mean. The 2017 trial results differed as both AB and R:B did not significantly respond to the WL regime application (Table 3).

Generally, plants subjected to cumulative water deficits (i.e., in tube and pot trials) may avert cell dehydration by decreasing the area of their transpiring AB and the rate of transpiration in the leaves (by decreasing stomatal conductance) and, thus, increasing WUE (Elazab et al., 2012; del Pozo et al., 2020). Other mechanisms such as maintaining high relative water content in the leaves (by accumulating solutes such as proline and soluble sugars) at levels close to WW plants and/or decreasing the osmotic potential could avoid cell dehydration under the WL regime (Acton, 2012).

The inconsistency between the 2016 and 2017 results could be due to the application to the WL regime different timing, period, and climatic conditions in the glasshouse trials. For the 2016 trial, the WL regime was applied for a short time from flag leaf expansion (Zadoks stage Z41) until anthesis (Z69), while in the 2017 trial, it was applied for a long time from tillering (Z26) until anthesis (Z69). However, the climatic conditions in the glasshouse during the 2016 trial encouraged the development of more severe water stress than the 2017 trial, where the air temperatures were almost 10°C higher and the relative humidity was almost 20% lower than those of the 2017 trial (Supplementary Table S3). Moreover, in the 2017 trial, the AB under both WW and WL regimes were higher than those of 2016 due to the longer growing period and the higher nutrient solution supplies (check the materials and methods). Thus, the WL plants in the 2016 trial reduced the area of their transpiring AB and the transpiration rate per plant leaf relative to the WW plants. On the other side, the WL plants in 2017 probably tended to adapt to the lower SWC by (1) maintaining high relative water content, (2) decreasing the osmotic potential, and (3) producing thinner roots, which are known to increase the water uptake area in the soil while slowing the water uptake rate from the root to the shoot (Palta et al., 2011). Thus, they were able to avert WL regime adverse effects by maintaining the production of AB similar to WW plants.

### Root Weight Density and Soil Water Content Distribution

A decrease in RWD from the upper to the lower depths of the soil profile is a well-known pattern of root distribution in wheat and other cereals (Gregory et al., 1978; Elazab et al., 2012, 2016; Liu et al., 2015; Aziz et al., 2017; Ali et al., 2018; Zhao et al., 2018). This gradient allocation of RWD is due to the presence of adventitious roots, which occupy the upper soil profile forming around 86–99% of the whole root system, and unlike seminal roots, they cannot grow in the deep soil profile layers (Manske and Vlek, 2002; Zhao et al., 2018). The recent study of Zhao et al. (2018) in winter wheat under optimal field conditions showed that 89.2% of the RWD in all growth stages (from seedling to maturity) was distributed in the upper soil profile (0–40 cm). Also, the increased soil strength in the soil profile’s deep layers is known to inhibit root growth and, thus, decreases RWD (Liu et al., 2015; Gao et al., 2016; Hodgkinson et al., 2017). All the genotypes evaluated under the WW and WL regimes in 2015, 2016, and 2017 showed this gradient distribution of RWD (Figures 1B, 2A,B,E,F), except for a slight increase in RWD in the 2017 trial in the lower soil layer for the genotype QUP2529 (in 140–160 cm) under the WW regime (Figure 2E). Previous studies have reported a slight increase in the RWD in the lower soil layers such as Elazab et al. (2012) for spring wheat under the WW regime, Gregory et al. (1978) in a winter wheat trial under WL field conditions, and Dwyer et al. (1988) in barley (*Hordeum vulgare* L.) in different locations with different soil type characteristics.

For both water regimes in the 2016 and 2017 trials, the SWC showed a gradient increase from the upper (0–20 cm) to the lower soil layers (120–140 cm) (Figures 2C,D,G,H and Supplementary Tables S1, S2). This gradient is a consequence of transpiration and drainage processes taking place simultaneously (Davidson et al., 1969; Carvalho, 2009; Braudeau and Mohtar, 2014). Thus, the SWC in the upper parts of the soil profile decreased due to plant transpiration, while the lower SWC increased due to vertical drainage of irrigation water in the tubes. Also, as mentioned previously, the existence of adventitious roots in the upper soil (i.e., the majority of root biomass) could exhaust most of the SWC in the upper soil profile. Similar patterns of SWC were reported by Liu et al. (2015) and Ali et al. (2018) for winter wheat in field conditions under different water regimes, regardless of the developmental stage (seedling, flowering, seed filling, and maturity).

It is believed that plants with extensive (i.e., high biomass allocation) deep root systems are more tolerant to water deficits than shallow-rooted plants (Elazab et al., 2016). The massive deep root system can access more considerable water resources from deeper layers of the soil profile and, thus, can take up additional soil resources for grain filling and thereby increasing final AB and GY (Nagesh, 2006; Ayad et al., 2010; Wasson et al., 2012; Wang et al., 2014; Elazab et al., 2016; Figueroa-Bustos et al., 2018). Thus, breeding programs for WL adaptation in seasonal rainfall regions, such as the Mediterranean basin, are focused on selecting plants with large root systems (Ma et al., 2010; Palta et al., 2011; Elazab et al., 2016).

The present study results in the 2016 and 2017 trials showed the importance of high biomass allocation to roots in the soil profile for more water extraction. In the 2016 trial, the WU and AB showed strong positive correlations with each other, and both showed a significant positive relationship with RWD distribution in the different soil profile depths under the WW and WL regimes (Table 4). Also, in 2017, WU was positively related to RWD under the WL regime (Table 4). However, as mentioned previously, no significant effect of the WL regime application was detected on both RB or RWD distribution in the different soil layers (Tables 2, 3 and Supplementary Tables S1, S2). Moreover, the results of SWC distributions under both water regimes in 2016 and under the WL regime in 2017 indicated a decrease in the SWC in the lowest soil depth (140–160 cm) with no comparable increase in the RWD of the same depth. However, this small root biomass was efficient at capturing water and decreased the SWC at that layer. This efficiency could be due to the production of thinner roots at that soil depth, which are known to produce a greater surface area per soil volume, and thereby the water uptake is enhanced (Bonifas and Lindquist, 2009; Elazab et al., 2016). In contrast, the QUP2529 genotype under the WW regime of the 2017 trial did not show the same root efficiency mechanism due to its small increase in RWD at the lowest soil layer (140–160 cm) (Figure 2G), where this increase was not translated to increased water uptake because the SWC at the same soil depth tended to increase. However, this access of the additional root biomass to water resources in the deep parts of the soil profile was enough to translate to a better leaf water status, as represented by reductions in the stable oxygen and hydrogen isotopic signatures and low evaporative enrichment in the genotype QUP2529 (Table 5).

Previous studies by several authors (Reynolds et al., 2007; Palta et al., 2011; Lynch et al., 2014; Vadez, 2014; Becker et al., 2016) reported that the increase in root biomass under water-deficit conditions is not necessarily an indication of higher AB or GY production. Moreover, Vadez (2014) reported that root biomass distribution is not necessarily crucial for more water uptake and other hydraulic biochemical (e.g., aquaporin’s activity) and anatomical (e.g., xylem vessel’s size) root traits are important for water uptake.

The production and subsequent maintenance of an extensive root system is a resource-exhausting process for the plant concerning assimilates required and respiration costs (Passioura, 1983; Ma et al., 2010; Elazab et al., 2016). A root system with the ability to take up limited soil resources at a lower metabolic expense would result in an increased WUE (Passioura, 1983; Ma et al., 2010; Lynch et al., 2014; Elazab et al., 2016). Production of thinner roots is an advantage in facilitating water and nutrient uptake under limited soil resources (Barraclough et al., 1989; Ryser, 1998; Bonifas and Lindquist, 2009; Corneo et al., 2016; Aziz et al., 2017) because it produces a larger root surface area per volume of soil and, thus, generates a greater surface area for resource uptake than in plants with thick root systems (Bonifas and Lindquist, 2009; Elazab et al., 2016). Furthermore, the metabolic expenses (production and maintenance of root tissues, measured in carbon units) of thinner root systems are lower than those of thick root systems (Cornelissen et al., 2003; Lynch et al., 2014; Elazab et al., 2016). Besides, thinner roots have a smaller diameter, which enables plants to decrease soil water uptake in the early period of the growth cycle and, thus, saves water for the reproduction stages (i.e., anthesis and grain filling) (Richards and Passioura, 1989; Condon et al., 2004; Bonifas and Lindquist, 2009; Palta et al., 2011; Vadez, 2014; Elazab et al., 2016). Production of thinner root systems in response to WL regimes has been reported previously for bread wheat growing in tubes (Nagesh, 2006; Becker et al., 2016) or pots (Song et al., 2010) and durum wheat and barley grown in tubes (Carvalho et al., 2014). Becker et al. (2016) reported that the increased length of thin roots in bread wheat positively correlates with both stomatal conductance and plant water use.

### Isotope Composition of the Soil and Plant Water Use

The analysis of δ^18^O and δ^2^H revealed the WL regime’s consistent significant effects on soil and stem water, but not in the leaves (Table 5). As expected, both the shallow and deep soil WCs were reduced in the WL regime, which was associated with isotopic enrichment in the deep soil layers due to evaporation (Table 5). The shallow soil seemed to be at the edge of the soil evaporation front, as suggested by its highly depleted isotope composition (lower δ^18^O and δ^2^H signatures), which is indicative of the diffusion of isotopically depleted water vapor from underlying soil layers in dry soils (Barnes and Allison, 1988; Palacio et al., 2014). Conversely, stem water was more depleted in WW than in WL plants, thus tracking the deep soil layers’ changes. Genotypes showed significant differences in leaf WC and leaf water isotopic composition (Table 5). The high positive correlations between ^18^O and ^2^H signatures and enrichments of the leaf under both WW and WL (Supplementary Figure S1) revealed that evaporative enrichment in the leaves is mainly due to plant transpiration and both ^18^O and ^2^H signals are coming from the same source of water (Epstein et al., 1977; Sternberg et al., 1984; Cernusak et al., 2016).

The genotype QUP2529 showed the highest leaf WC, the lowest leaf δ^18^O and δ^2^H values, and the lowest evaporative enrichment (Δ^18^O, Δ^2^H). These results can be attributed to lower leaf temperatures and higher transpiration rates (Farquhar and Gan, 2003; Sheshshayee et al., 2005; Cabrera-Bosquet et al., 2009; Ferrio et al., 2009, 2012). The response of QUP2529 partly relies on more effective water uptake, which would allow this genotype to maintain higher transpiration rates under mild water stress. Our results are in agreement with the previous studies done by Lopes and Reynolds (2010) and Becker et al. (2016) in synthetic hexaploid-derived wheat lines, where they reported that under water deficit, the increase in root biomass allocation to the deepest soil layer was associated with a cooler canopy temperature (a proxy measure indicating better water status) and enhanced water uptake.

Although significant differences were detected in deep soil and stem water isotope composition between the WW and WL regimes (Table 5), the Bayesian mixing models did not efficiently predict the plant’s primary water sources (shallow or deep soil water). Considerable uncertainty of the models was detected in the WL regime (Figures 3B,D) and was likely due to partial evaporation of stem water in the most stressed individuals (values in the top right corner in Figure 3D). However, the models were able to depict the larger relative contribution of deep soil WC in WL plants, particularly for cv. Pantera-INIA (Figure 3D). Also, the genotype QUP2569 had the lowest deep soil water contribution under the WW regime (Figure 3C).

## Conclusion

The present study showed the importance of high RWD distribution in soil depths to increase the WU and AB, especially under the WL regime in both the 2016 and 2017 trials. However, unlike the common belief that an extensive root system is advantageous under WL conditions, the root biomass under the WL regime did not differ significantly from the WW regime plants. The present study highlighted the role of a more efficient root system (Figure 2, Table 4, and Supplementary Tables S1, S2), rather than an extensive root system (i.e., more biomass allocation to roots), as a strategy to improve resource uptake under soil-limited water conditions at a decreased metabolic expense. Moreover, an extensive root system under WL regimes could cause rapid soil moisture consumption early in the growth cycle before the plant reaches the reproductive stages.

The root architecture and the stable isotope compositions and enrichment of the leaves explained the water status of the genotype QUP2529, where the genotype increased its RWD in lower soil depths (140–160 cm), and thus, it accessed more water resources, which consequently increased the plant transpiration and maintained better water status as expressed by the lowest leaf δ^18^O, δ^2^H, Δ^18^O, and Δ2H.

Significant differences were detected in deep soil and stem water isotope composition between the WW and WL regimes. However, the Bayesian mixing models did not efficiently predict the plant’s primary water sources (shallow or deep soil water).

## Data Availability Statement

The original contributions presented in the study are included in the article/Supplementary Material, further inquiries can be directed to the corresponding author.

## Author Contributions

AP and NB-S designed the experiments. NB-S performed all the glasshouse experiments and root evaluations. JF undertook the isotope analyses. MO and AE performed the statistical analysis. AE and AP were in charge of the writing, but all the authors contributed to the manuscript. All authors read and approved the final manuscript.

## Conflict of Interest

The authors declare that the research was conducted in the absence of any commercial or financial relationships that could be construed as a potential conflict of interest.
