# Supplementary material for: Root Architecture and Functional Traits of Spring Wheat Under Contrasting Water Regimes
Source: Front Plant Sci. 2020 Nov 11;11:581140. doi: 10.3389/fpls.2020.581140 (PMC7686047; doi:10.3389/fpls.2020.581140)
Supplement: Supplementary file 1 [file Table_1.DOCX]

**TABLE S1**. Genotypic mean values of root weight density (RWD, g m^-3^) and soil water content (Ө, cm^3^ cm^-3^) studied under different water regimes in the 2016 trials.

| **Trait** |  | **Mean (HSD-Tukey)** | | | | | | | | | | |  | **Mean** | |
| --- | --- | --- | --- | --- | --- | --- | --- | --- | --- | --- | --- | --- | --- | --- | --- |
|  |  | **WW** | | | | |  | **WL** | | | | |  |  |  |
|  |  | **Pantera-INIA** | **QUP2569** | **FONTAGRO98** | **QUP2529** | **FONTAGRO8** |  | **Pantera-INIA** | **QUP2569** | **FONTAGRO98** | **QUP2529** | **FONTAGRO8** |  | **WW** | **WL** |
| RWD1 |  | 252.69 ab | 426.97 c | 322.55 b | 277.01 ab | 191.78 a |  | 200.01 a | 312.06 b | 246.18 a | 307.65 b | 199.98 a |  | 294.20 | 253.18 |
| RWD2 |  | 101.78 a | 186.73 b | 122.42 a | 106.95 a | 125.36 a |  | 97.40 | 136.00 | 107.11 | 109.45 | 112.63 |  | 128.65 | 112.52 |
| RWD3 |  | 69.93 a | 175.85 b | 131.24 ab | 109.20 a | 112.88 ab |  | 104.08 ab | 159.84 b | 95.61 a | 115.61 ab | 102.39 ab |  | 119.82 | 115.51 |
| RWD4 |  | 85.37 ab | 146.81 b | 115.22 ab | 119.83 ab | 81.30 a |  | 86.27 | 138.75 | 87.94 | 108.49 | 77.30 |  | 109.71 | 99.75 |
| RWD5 |  | 34.82 a | 104.74 b | 72.73 ab | 76.45 b | 33.51 a |  | 76.80 | 81.87 | 65.92 | 87.86 | 56.35 |  | 64.45 | 73.76 |
| RWD6 |  | 19.35 ab | 58.74 c | 35.22 abc | 45.69 bc | 18.58 a |  | 37.58 | 44.99 | 53.64 | 59.31 | 42.62 |  | 35.52 | 47.63 |
| RWD7 |  | 10.15 a | 29.25 b | 16.82 ab | 29.73 b | 11.39 a |  | 9.81 | 21.89 | 29.64 | 37.62 | 24.45 |  | 19.47 | 24.68 |
| RWD8 |  | 4.89 | 10.53 | 6.94 | 11.16 | 4.17 |  | 7.74 | 5.13 | 11.47 | 14.05 | 6.67 |  | 7.54 | 9.01 |
| Ө 1 |  | 0.07 | 0.11 | 0.08 | 0.14 | 0.06 |  | 0.04 | 0.03 | 0.03 | 0.04 | 0.07 |  | **0.09** | **0.04** |
| Ө 2 |  | 0.10 a | 0.12 ab | 0.11 a | 0.16 ab | 0.18 b |  | 0.04 | 0.04 | 0.04 | 0.05 | 0.06 |  | **0.14** | **0.05** |
| Ө 3 |  | 0.19 | 0.15 | 0.14 | 0.16 | 0.25 |  | 0.05 | 0.04 | 0.05 | 0.04 | 0.07 |  | **0.18** | **0.05** |
| Ө 4 |  | 0.21 | 0.18 | 0.20 | 0.17 | 0.24 |  | 0.08 ab | 0.05 a | 0.07 ab | 0.05 a | 0.09 b |  | **0.20** | **0.07** |
| Ө 5 |  | 0.24 | 0.23 | 0.23 | 0.23 | 0.26 |  | 0.11 | 0.09 | 0.10 | 0.05 | 0.13 |  | **0.24** | **0.10** |
| Ө 6 |  | 0.26 ab | 0.26 ab | 0.25 a | 0.26 ab | 0.28 b |  | 0.17 b | 0.13 ab | 0.14 ab | 0.08 a | 0.19 b |  | **0.26** | **0.14** |
| Ө 7 |  | 0.26 | 0.27 | 0.28 | 0.26 | 0.31 |  | 0.23 b | 0.16 ab | 0.16 ab | 0.09 a | 0.16 ab |  | **0.28** | **0.16** |
| Ө 8 |  | 0.18 | 0.13 | 0.17 | 0.11 | 0.14 |  | 0.11 ab | 0.08 ab | 0.09 ab | 0.04 a | 0.12 b |  | **0.15** | **0.09** |

The number following the acronym of the trait refers to the soil column depth where the trait was measured: 1 refers to 0-20 cm; 2 refers to 20-40 cm; 3 refers to 40-60 cm; 4 refers to 60-80 cm; 5 refers to 80-100 cm; 6 refers to 100-120 cm; 7 refers to 120-140 cm; and 8 refers to 140-160 cm. Genotypes means followed by different letters were significantly different (*P* *≤* 0.05) by Tukey’s HSD test. WW, well-watered plants; and WS, water-limited plants. For the WW and WL genotypes means, bold numbers represent significant differences according to the ANOVA analysis.
