# Supplementary material for: Root Architecture and Functional Traits of Spring Wheat Under Contrasting Water Regimes
Source: Front Plant Sci. 2020 Nov 11;11:581140. doi: 10.3389/fpls.2020.581140 (PMC7686047; doi:10.3389/fpls.2020.581140)
Supplement: Supplementary file 2 [file Table_2.DOCX]

**TABLE S2**. Genotypic mean values of root weight density (RWD, g m^-3^) and soil water content (Ө, cm^3^ cm^-3^) studied under different water regimes in the 2017 trials.

| **Trait** |  | **Mean (HSD-Tukey)** | | | | | | | | | | |  | **Mean** | |
| --- | --- | --- | --- | --- | --- | --- | --- | --- | --- | --- | --- | --- | --- | --- | --- |
|  |  | **WW** | | | | |  | **WL** | | | | |  |  |  |
|  |  | **Pantera-INIA** | **QUP2569** | **FONTAGRO98** | **QUP2529** | **FONTAGRO8** |  | **Pantera-INIA** | **QUP2569** | **FONTAGRO98** | **QUP2529** | **FONTAGRO8** |  | **WW** | **WS** |
| RWD1 |  | 161.71 a | 351.94 c | 226.56 b | 240.27 b | 185.01 a |  | 118.45 | 207.32 | 200.20 | 197.02 | 214.07 |  | 233.10 | 187.41 |
| RWD2 |  | 75.13 a | 146.05 b | 99.12 ab | 115.91 ab | 110.42 ab |  | 73.50 a | 118.53 b | 90.74 ab | 89.08 ab | 104.62 ab |  | 109.33 | 95.29 |
| RWD3 |  | 77.36 | 138.83 | 92.50 | 111.52 | 128.82 |  | 61.35 | 92.36 | 80.49 | 68.85 | 101.64 |  | **109.81** | **80.94** |
| RWD4 |  | 84.80 | 142.51 | 92.07 | 91.40 | 91.76 |  | 62.52 | 94.46 | 70.27 | 54.30 | 103.83 |  | 100.51 | 77.08 |
| RWD5 |  | 64.03 | 101.62 | 83.51 | 100.62 | 101.45 |  | 53.36 | 116.39 | 76.34 | 94.71 | 94.57 |  | 90.25 | 87.08 |
| RWD6 |  | 80.56 | 96.11 | 60.16 | 76.63 | 72.00 |  | 51.36 | 111.27 | 82.30 | 97.68 | 92.34 |  | 77.09 | 86.99 |
| RWD7 |  | 34.12 | 91.60 | 60.46 | 43.41 | 80.22 |  | 26.84 a | 75.49 ab | 72.22 ab | 77.70 ab | 112.09 b |  | 61.96 | 72.87 |
| RWD8 |  | 39.29 a | 69.23 ab | 59.02 ab | 84.17 b | 47.22 a |  | 16.45 | 57.45 | 56.43 | 60.79 | 38.67 |  | 59.79 | 45.96 |
| Ө 1 |  | 0.08 | 0.07 | 0.08 | 0.06 | 0.06 |  | 0.06 | 0.07 | 0.04 | 0.03 | 0.02 |  | **0.07** | **0.05** |
| Ө 2 |  | 0.10 b | 0.06 a | 0.08 ab | 0.07 a | 0.08 ab |  | 0.07 | 0.04 | 0.04 | 0.05 | 0.05 |  | **0.08** | **0.05** |
| Ө 3 |  | 0.12 | 0.06 | 0.08 | 0.09 | 0.10 |  | 0.09 | 0.06 | 0.06 | 0.06 | 0.06 |  | **0.09** | **0.07** |
| Ө 4 |  | 0.11 | 0.10 | 0.11 | 0.14 | 0.12 |  | 0.10 | 0.08 | 0.06 | 0.08 | 0.08 |  | **0.12** | **0.08** |
| Ө 5 |  | 0.13 | 0.12 | 0.13 | 0.14 | 0.15 |  | 0.14 | 0.06 | 0.08 | 0.09 | 0.09 |  | **0.13** | **0.09** |
| Ө 6 |  | 0.21 | 0.15 | 0.13 | 0.17 | 0.19 |  | 0.17 b | 0.05 a | 0.08 a | 0.11 ab | 0.09 a |  | **0.17** | **0.10** |
| Ө 7 |  | 0.24 | 0.20 | 0.19 | 0.21 | 0.20 |  | 0.21 b | 0.07 a | 0.10 ab | 0.12 ab | 0.14 ab |  | **0.21** | **0.13** |
| Ө 8 |  | 0.19 b | 0.21 b | 0.20 b | 0.23 b | 0.11 a |  | 0.10 | 0.05 | 0.09 | 0.10 | 0.05 |  | **0.19** | **0.08** |

The number following the acronym of the trait refers to the soil column depth where the trait was measured: 1 refers to 0-20 cm; 2 refers to 20-40 cm; 3 refers to 40-60 cm; 4 refers to 60-80 cm; 5 refers to 80-100 cm; 6 refers to 100-120 cm; 7 refers to 120-140 cm; and 8 refers to 140-160 cm. Genotypes means followed by different letters were significantly different (*P* *≤* 0.05) by Tukey’s HSD test. WW, well-watered plants; and WL, water-limited plants. For the WW and WL genotypes means, bold numbers represent significant differences according to the ANOVA analysis.
