# Supplementary material for: Root Architecture and Functional Traits of Spring Wheat Under Contrasting Water Regimes
Source: Front Plant Sci. 2020 Nov 11;11:581140. doi: 10.3389/fpls.2020.581140 (PMC7686047; doi:10.3389/fpls.2020.581140)
Supplement: Supplementary file 3 [file Table_3.DOCX]

**TABLE S3**. Mean monthly maximum (Max), average (Avr), and minimum (Min) temperatures; and mean monthly maximum (Max), average (Avr), and minimum (Min) relative humidity (RH %) at the greenhouse trial in Talca, Chile.

| a) Month-2015 | ºC Max | ºC Avr | ºC Min | RH % Max | RH % Avr | RH % Min |
| --- | --- | --- | --- | --- | --- | --- |
| Aug | 22.20 | 15.14 | 11.40 | 70.20 | 52.10 | 34.00 |
| Sep | 23.90 | 15.94 | 11.30 | 69.10 | 48.40 | 27.60 |
| Oct | 27.10 | 18.24 | 12.70 | 68.00 | 41.20 | 14.40 |
| Nov | 31.30 | 21.64 | 15.20 | 65.60 | 38.40 | 11.20 |
|  |  |  |  |  |  |  |
| b) Month-2016 | ºC Max | ºC Avr | ºC Min | RH % Max | RH % Avr | RH % Min |
| Sep | 33.20 | 20.40 | 10.20 | 78.90 | 51.70 | 24.40 |
| Oct | 34.40 | 22.30 | 12.80 | 78.10 | 50.60 | 23.00 |
| Nov | 39.20 | 25.60 | 14.70 | 74.80 | 45.10 | 15.40 |
|  |  |  |  |  |  |  |
| c) Month-2017 | ºC Max | ºC Avr | ºC Min | RH % Max | RH % Avr | RH % Min |
| May | 21.48 | 13.74 | 9.02 | 88.51 | 77.03 | 53.93 |
| Jun | 18.88 | 11.97 | 7.78 | 92.31 | 84.84 | 67.78 |
| Jul | 19.44 | 10.29 | 4.90 | 95.68 | 86.42 | 62.31 |
| Aug | 22.47 | 13.34 | 7.60 | 88.91 | 74.13 | 47.78 |
| Sep | 30.59 | 17.99 | 9.64 | 76.69 | 55.05 | 28.01 |
