# Supplementary material for: Root Architecture and Functional Traits of Spring Wheat Under Contrasting Water Regimes
Source: Front Plant Sci. 2020 Nov 11;11:581140. doi: 10.3389/fpls.2020.581140 (PMC7686047; doi:10.3389/fpls.2020.581140)
Supplement: Supplementary file 4 [file Table_4.DOCX]

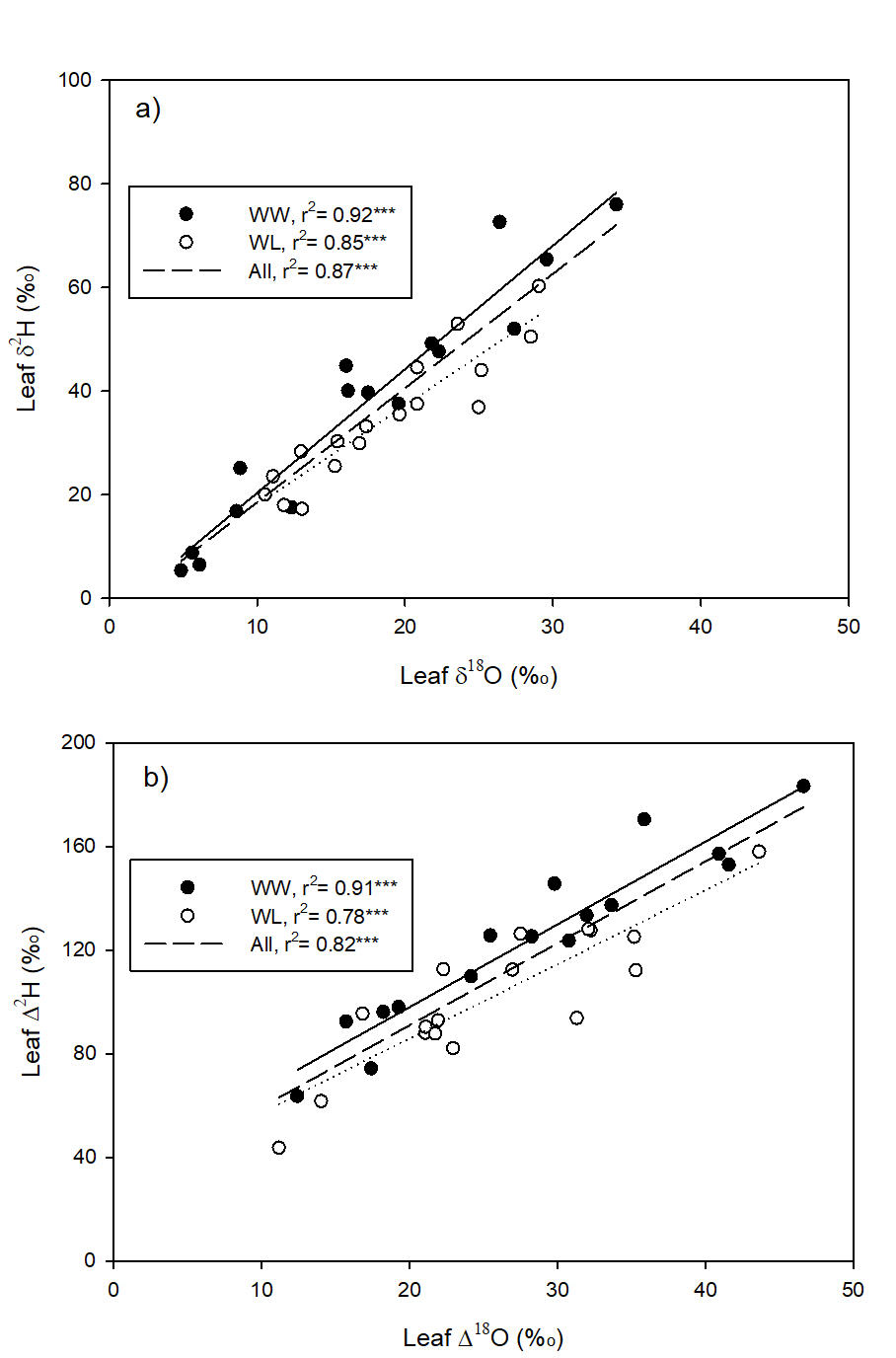


**FIGURE S1.** Linear regression of the relationship between: the leaf oxygen (δ^18^O) and hydrogen (δ^2^H) isotope compositions (a); and the leaf water oxygen (Δ^18^O) and hydrogen (Δ^2^H) isotopic enrichment above source water (b). Closed circles, WW regime; and open circles, WS regime. Level of significance: ***, *P* ≤ 0.001.
